# Wildlife Is a Potential Source of Human Infections of *Enterocytozoon bieneusi* and *Giardia duodenalis* in Southeastern China

**DOI:** 10.3389/fmicb.2021.692837

**Published:** 2021-08-10

**Authors:** Yan Zhang, Rongsheng Mi, Lijuan Yang, Haiyan Gong, Chunzhong Xu, Yongqi Feng, Xinsheng Chen, Yan Huang, Xiangan Han, Zhaoguo Chen

**Affiliations:** ^1^Key Laboratory of Animal Parasitology of Ministry of Agriculture, Laboratory of Quality and Safety Risk Assessment for Animal Products on Biohazards (Shanghai) of Ministry of Agriculture, Shanghai Veterinary Research Institute, Chinese Academy of Agricultural Sciences, Shanghai, China; ^2^Shanghai Wild Animal Park, Shanghai, China

**Keywords:** *Cryptosporidium*, *Enterocytozoon bieneusi*, *Giardia duodenalis*, genotypes, wildlife, prevalence, zoonotic potential

## Abstract

Wildlife is known to be a source of high-impact pathogens affecting people. However, the distribution, genetic diversity, and zoonotic potential of *Cryptosporidium*, *Enterocytozoon bieneusi*, and *Giardia duodenalis* in wildlife are poorly understood. Here, we conducted the first molecular epidemiological investigation of these three pathogens in wildlife in Zhejiang and Shanghai, China. Genomic DNAs were derived from 182 individual fecal samples from wildlife and then subjected to a nested polymerase chain reaction–based sequencing approach for detection and characterization. Altogether, 3 (1.6%), 21 (11.5%), and 48 (26.4%) specimens tested positive for *Cryptosporidium* species, *E. bieneusi*, and *G. duodenalis*, respectively. Sequence analyses revealed five known (BEB6, D, MJ13, SC02, and type IV) and two novel (designated SH_ch1 and SH_deer1) genotypes of *E. bieneusi*. Phylogenetically, novel *E. bieneusi* genotype SH_deer1 fell into group 6, and the other genotypes were assigned to group 1 with zoonotic potential. Three novel *Cryptosporidium* genotypes (*Cryptosporidium* avian genotype V-like and *C. galli*-like 1 and 2) were identified, *C. galli*-like 1 and 2 formed a clade that was distinct from *Cryptosporidium* species. The genetic distinctiveness of these two novel genotypes suggests that they represent a new species of *Cryptosporidium.* Zoonotic assemblage A (*n* = 36) and host-adapted assemblages C (*n* = 1) and E (*n* = 7) of *G. duodenalis* were characterized. The overall results suggest that wildlife act as host reservoirs carrying zoonotic *E. bieneusi* and *G. duodenalis*, potentially enabling transmission from wildlife to humans and other animals.

## Introduction

Wildlife has been an important source of various high-impact pathogens affecting people, and zoonoses originated in wildlife remain a major public health issue around the world (Kruse et al., 2005). Novel diseases continue to emerge, and the responsible pathogens are often from unexpected wildlife, such as Ebola and Marburg virus (Bats; Amman et al., 2015; Jones et al., 2015); HIV-1 and HIV-2 (Primates; Gao et al., 1999); Nipah, Hendra, and Menangle virus (Bats; Field et al., 2007); West Nile virus (Mosquitoes; CDC, 1999, 2000); SARS (severe acute respiratory syndrome)–like virus (Bats; Li et al., 2005); and 2019 novel coronavirus (COVID-19) (Wildlife; Liu et al., 2020; Xiao et al., 2020), highlighting the important role of wildlife in the transmission of zoonotic pathogens. Currently, the ongoing 2019 novel coronavirus pandemic has resounded the alarm on pathogens in wildlife. Thus, it is crucial to screen and identify potentially zoonotic pathogens in wildlife from different geographical regions for prediction, prevention, and control of zoonotic diseases outbreaks in humans (Cunningham et al., 2017).

Infectious diarrhea remains a major public health concern worldwide (Walker et al., 2012; Liu et al., 2015; GBD, 2016). It kills more than 2,000 children every day, more than AIDS, malaria, and measles (Liu et al., 2015; Chingwaru and Vidmar, 2018; Lemos et al., 2018; Maniga et al., 2018). Multiple pathogens including viruses, bacteria (Blander et al., 2017), fungi (Liguori et al., 2015; Hallen-Adams and Suhr, 2017), and protists (Lanata et al., 2013; Blander et al., 2017) are responsible for diarrhea. Among protists, *Cryptosporidium* species, *Giardia duodenalis*, and *Enterocytozoon bieneusi* are the most common etiological pathogens of the intestinal disease and are known to cause large disease outbreaks in humans, especially for *Cryptosporidium* and *G. duodenalis* (Karanis et al., 2007; Baldursson and Karanis, 2011; Decraene et al., 2012; Ryan and Cacciò, 2013; Checkley et al., 2015).

Currently, ∼40 named *Cryptosporidium* species and close to 50 genotypes have been reported (Feng et al., 2018; Haghi et al., 2020). There are ∼20 species of *Cryptosporidium* identified in humans (Xiao, 2010), of which *Cryptosporidium parvum* and *Cryptosporidium hominis* are the most common species infecting humans (Feng et al., 2018). *C. parvum* has a broad host range that includes humans and various animal species. By contrast, *C. hominis* is mainly restricted to humans, non-human primates, and equine animals (Feng et al., 2018). *G. duodenalis* is recognized as a species complex consisting of eight assemblages (A–H). Assemblages A and B can infect humans and other mammals, assemblages C and D are frequently found in dogs and other canids, assemblage E in hoofed animals, assemblage F in cats, assemblage G in rodents, and assemblage H in pinnipeds (Ryan and Zahedi, 2019). Until recently, assemblages C to H were considered host-specific, except that assemblages C, D, E, and F are occasionally found in humans (i.e., assemblage E has been found in human samples more frequently than F) (Gelanew et al., 2007; Broglia et al., 2013; Liu et al., 2014; Štrkolcová et al., 2015; Scalia et al., 2016; Zahedi et al., 2017). Among 14 species of microsporidia infecting humans, *E. bieneusi* is the most common microbe causing diarrhea. *E. bieneusi* can infect a broad host range, including mammals, birds, reptiles (Squamata), and insects (Diptera). Currently, there are more than 600 genotypes, and most genotypes can be found in both humans and animals, showing zoonotic potential (Li et al., 2019a, b; Zhang, 2019; Zhang et al., 2021). The three enteric eukaryotic agents can infect humans through the fecal–oral route, via direct contact with infected individuals or ingestion of contaminated water or food (Yu et al., 2020).

The three microbes can be identified or characterized at species, subspecies, and/or genotypic level using molecular techniques. Currently, small subunit ribosomal DNA (*SSU* rDNA) has been wildly used for *Cryptosporidium* species identification, whereas a genetic marker in the 60-kDa glycoprotein (*gp60*) gene has been commonly used for differentiating *Cryptosporidium* at the genotypic and subgenotypic levels (Abeywardena et al., 2015). For *G. duodenalis*, triose-phosphate isomerase (*tpi*), β-giardin (*bg*), glutamate dehydrogenase (*gdh*), elongation factor 1-alfa (*ef1-*α), and *SSU* rDNA are commonly used for genotypic identification (Ryan and Cacciò, 2013). As *ef1-*α and *SSU* rDNA are relatively problematic, and they cannot discriminate *G. duodenalis* subtypes within assemblages accurately and are thus not useful for transmission analyses (Traub et al., 2004). Internal transcribed spacer (ITS) of nuclear ribosomal DNA is sufficiently variable for the identification and genotypic characterization of *E. bieneusi* (Santín et al., 2009).

Using the approach above, we have explored the microbes from various animals including wild deer (Zhang et al., 2018b; Koehler et al., 2020), marsupials (Zhang et al., 2018c), domestic alpacas (Koehler et al., 2018), cattle (Zhang et al., 2018a), goats and sheep (Zhang et al., 2020), companion cats and dogs (Zhang et al., 2019), and humans (Zhang et al., 2018e). We also established a new phylogenetic classification system of overall 600 *E. bieneusi* genotypes (Zhang et al., 2021). The present study aims to identify three pathogens (*Cryptosporidium* species, *E. bieneusi*, and *G. duodenalis*) in wildlife in Zhejiang and Shanghai, characterize their genotypes and analyze their zoonotic potential. The findings in this study would help to understand the genetic diversity of the three agents and provide critical information for future global strategies to prevent outbreaks of their zoonoses.

## Materials and Methods

### Samples and DNA Isolation

In total, 182 fecal samples were collected from 48 species of zoo animals from Zhejiang zoo (*n* = 52) and Shanghai Wild Animal Park (*n* = 130) from May 2018 to August 2020 (Supplementary Table 1). Some fecal samples were collected from wildlife rectum directly, whereas others were fresh deposited fecal samples. Genomic DNA was extracted directly from 0.1 to 0.4 g of each of the 182 fecal samples using the FastDNA SPIN Kit for Soil (MP Biomedicals, Santa Ana, CA, United States) according to the manufacturer’s recommendations. The extracted DNA was stored at −20°C for further polymerase chain reaction (PCR) assay.

### Detection of *Cryptosporidium* Species, *E. bieneusi* and *G. duodenalis*

#### Nested PCR-Based Sequencing of *Cryptosporidium* Species *SSU* rDNA

The small subunit of ribosomal nuclear DNA locus (target length 830 bp) of each sample was screened for identification of *Cryptosporidium* species (i.e., primers are listed in Table 1). In the first run, PCR contained 25 μL of 2 × PCR buffer for KOD FX (Mg^2+^ plus) (Toyobo, Japan), 2 mM dNTPs, 100 nM (each) primer, 1.0 U KOD FX, and 1 μL of DNA template in a total 50 μL reaction mixture. A total of 35 cycles were carried out, each consisting of 94°C for 45 s, 55°C for 45 s, and 72°C for 1 min, with an initial hot start at 94°C for 3 min, and a final extension at 72°C for 7 min. A secondary PCR product was then amplified from 2 μL of the primary PCR products with the same cycling conditions as the first run, except for 60°C annealing temperature (Xiao et al., 1999, 2001; Jiang et al., 2005).

**TABLE 1:** PCR primers (forward and reserve) used for the amplification of *Cryptosporidium*, *Enterocytozoon bieneusi*, and *Giardia duodenalis* in this study.

Species (genetic marker)	Primers (5′-3′)	Length (∼bp)	References
*Cryptosporidium*	TTC TAG AGC TAA TAC ATG CG	1,325	Xiao et al., 1999
(*SSU* rDNA)	CCC ATT TCC TTC GAA ACA GGA		Xiao et al., 2001
	GGA AGG GTT GTA TTT ATT AGA TAA AG	830	Jiang et al., 2005
	CTC ATA AGG TGC TGA AGG AGT A		
*E. bieneusi*	MSP-1 (TGA ATG KGT CCC TGT)	590	Katzwinkel-Wladarsch et al., 1996
(ITS)	MSP-2B (GTT CAT TCG CAC TAC T)		
	MSP-3 (GGA ATT CAC ACC GCC CGT CRY TAT)	508	
	MSP-4B (CCA AGC TTA TGC TTA AGT CCA GGG AG)		
*G. duodenalis*	AL3543 (AAA TTA TGC CTG CTC GTC G)	605	Sulaiman et al., 2003
(*tpi*)	AL3546 (CAA ACC TTT TCC GCA AAC C)		
	AL3544 (CCC TTC ATC GGT GGT AAC TT)	532	
	AL3545 (GTG GCC ACC ACT CCC GTG CC)		

#### Nested PCR-Based Sequencing of *E. bieneusi* ITS

Individual genomic DNA samples were subjected to nested PCR-coupled sequencing of the *ITS* (243–245 bp) region (i.e., only 243–245-bp fragment of the ITS was used for further phylogenetic analyses) using an established technique (Katzwinkel-Wladarsch et al., 1996). Nested PCR (in 50 μL) was conducted in a standard buffer containing 3.0 μM MgCl_2_, 0.4 mM dNTPs, 50 pmol of each primer, 1.25 U of Ex Taq DNA (TaKaRa Bio Inc., Beijing, China), and DNA template—except for the negative (no-template) control. The cycling conditions for both primary and secondary (nested) PCRs were as follows: 94°C for 5 min (initial denaturation), followed by 35 cycles of 94°C for 45 s (denaturation), 54°C for 45 s (annealing), and 72°C for 1 min (extension), followed by 72°C for 10 min (final extension).

#### Nested PCR-Based Sequencing of *G. duodenalis TPI*

*G. duodenalis* assemblages were identified and characterized by nested PCR-based sequencing of the *tpi* gene (∼530 bp) using the established methods (Sulaiman et al., 2003). PCR was carried out in a volume of 50 μL containing 3.0 μM MgCl_2_, 0.4 mM dNTPs, 50 pmol of each primer, 1.25 U of Ex Taq DNA (TaKaRa Bio Inc., Beijing, China), and DNA template. A cycling protocol of 94°C for 5 min (initial denaturation), followed by 35 cycles of 94°C for 45 s (denaturation), 50°C for 45 s (annealing), 72°C for 1 min (extension), and a final extension of 72°C for 10 min. The secondary amplification was achieved using the same cycling conditions, except for the annealing temperature of 55°C for 30 s.

Known test-positive, test-negative, and no-template controls were included in each PCR run. The secondary PCR products were examined by gel electrophoresis on a 1.5% agarose gel containing 4S Green Plus Nucleic Acid Stain (Sangon Biotech, Shanghai, China) and directly sequenced using second-round PCR primers in both directions. All sequences obtained (GenBank accession nos. *Cryptosporidium*: MW168840-MW168842; *E. bieneusi*: MT895455-MT895461 and *G. duodenalis*: MW048593-MW048601) were inspected for quality and compared with reference sequences acquired from the GenBank database.

### Phylogenetic Analysis

Obtained sequences from this and previous studies were aligned over a consensus length of 735 (*Cryptosporidium*), 459 (*G. duodenalis*), and 270 (*E. bieneusi;* after trimming, approximately 243-bp fragment of the ITS was analyzed) positions using previously established methods (Zhang et al., 2018d) and then subjected to phylogenetic analyses using the Bayesian inference (BI) and Monte Carlo Markov Chain methods in MrBayes v.3.2.3 (Huelsenbeck and Ronquist, 2001). The Akaike Information Criteria test in jModeltest v.2.1.7 (Darriba et al., 2012) was used to evaluate the likelihood parameters set for BI analysis. Posterior probability (*pp*) values were calculated by running 2,000,000 generations with four simultaneous tree-building chains, with trees saved every one-hundredth generation. A 50% majority-rule consensus tree for each analysis was constructed based on the final 75% of trees generated by BI. The clades and subclades were assigned and named using an established classification system (Santín and Fayer, 2009, 2011; Feng and Xiao, 2011; Karim et al., 2015; Li W. et al., 2015; Koehler et al., 2016; Li et al., 2019a, b; Ryan and Zahedi, 2019).

## Results

### Molecular Detection of *Cryptosporidium* Species Based on *SSU* rDNA Gene

In total, three fecal DNA samples were identified *Cryptosporidium* species with the prevalence of 1.6% (3/182) (Table 2). They were all novel *SSU* rDNA sequences (i.e., < 100% identity with a sequence on GenBank) uniquely form the zoo in Zhejiang (Table 3). The three novel *SSU* rDNA sequences were assigned to the most closely related species or genotypes of *Cryptosporidium* based on sequence identity, representing *Cryptosporidium galli*-like 1 (from a species of Psittacidae) and *C. galli*-like 2 (channel-billed toucan) and *Cryptosporidium* avian genotype V-like (green aracari). *C. galli*-like 1 and 2 differed by 18 bp (763/781; 97.7%) and 17 bp (765/778; 98.3%) from the sequences representing *C. galli* (GenBank accession no. MG516766), and *Cryptosporidium* avian genotype V-like differed by 7 bp (780/787; 99.1%) from a sequence with GenBank accession no. JX548292 (*Cryptosporidium* avian genotype V).

**TABLE 2:** Prevalence of *Cryptosporidium* species, *Enterocytozoon bieneusi*, and *Giardia duodenalis* in Shanghai Wild Animal Park and Zhejiang zoo of China.

Species	Prevalence of each species (%)	Total no. of positive/total no. of samples	Prevalence in each region (%) (no. of positive/no. of samples)
		
		Shanghai	Zhejiang
*Cryptosporidium*	1.6 3/182	0	1.6 (3/52)
*E. bieneusi*	11.5 21/182	14.6 (19/130)	3.8 (2/52)
*G. duodenalis*	26.4 48/182	30.8 (40/130)	15.4 (8/52)

**TABLE 3:** Summary of all pathogen species, genotypes, and/or assemblages identified in wildlife in Zhenjiang and Shanghai, China, using PCR-based sequencing of particular genetic markers.

Species/genotype/assemblage identified by PCR based on sequencing (positivity no.)	Genetic marker used	GenBank accession no.	Host (Latin name)	Positivity no. for each wild animal species
*Cryptosporidium* species V-like	(1) *SSU*	MW168842*	Green aracari (*Pteroglossus viridis*)	(1)
*C. galli*-like 1	(2) *SSU*	MW168841*	Psittacidae (species unknown)	(1)
*C. galli*-like *2*		MW168840*	Channel-billed toucan (*Ramphastos vitellinus*)	(1)
*Giardia duodenalis* A	(40) *tpi*	MW048593	Alpaca (*Vicugna pacos*)	(2)
		MW048598*	Siberian tiger (*Panthera tigris altaica*)	(2)
		MW048599*	Black-necked Crane (*Grus nigricollis*)	(2)
		MW048600*	Blue-headed macaw (*Propyrrhura couloni*)	(3)
		MW048601*	Cheetah (*Acinonyx jubatus*)	(3)
		MW048594^a^	Fennec fox (*Vulpes zerda*)	(2)
		MW048595^a^	Giant Eland (*Tragelaphus derbianus*)	(1)
		MW048596^a^	Giraffe (*Giraffa camelopardalis*)	(3)
		MW048597^a^	Golden takin (*Budorcas taxicolor bedfordi*)	(1)
			Great pied hornbill (*Buceros bicomis*)	(1)
			Hippopotamus (*Hippopotamus amphibious*)	(1)
			Lion (*Panthera leo*)	(2)
			Malabar pied hornbill (*Anthracoceros coronatus*)	(1)
			Snub-nosed monkey (*Rhinopithecus roxellana*)	(8)
			Ostrich (*Struthio camelus*)	(2)
			Peafowl (*Pavo cristatus*)	(2)
			Scarlet macaw (*Ara macao*)	(1)
			Sika deer (*Cervus Nippon*)	(1)
			Sun parakeet (*Aratinga solstitialis*)	(1)
			Tiger (*Panthera tigris tigris*)	(1)
*G. duodenalis* C	(1) *tpi*	MW048604*	Spotted hyena (*Crocuta crocuta*)	(1)
*G. duodenalis* E	(5) *tpi*	MW048602	Giraffe (*Giraffa camelopardalis*)	(4)
			Kangaroo (*Macropus* species)	(1)
	(2) *tpi*	MW048603*	Giraffe (*Giraffa camelopardalis*)	(2)
*Enterocytozoon bieneusi* BEB6	(3) ITS	MT895455	Alpaca (*Vicugna pacos*)	(1)
			Fallow deer (*Dama dama*)	(1)
			Red deer (*Cervus elaphus*)	(1)
*E. bieneusi* D	(8) ITS	MT895457	Siberian tiger (*Panthera tigris altaica*)	(2)
			Lion (*Panthera leo*)	(2)
			Snub-nosed monkey (*Rhinopithecus roxellana*)	(2)
			Tiger (*Panthera tigris tigris*)	(2)
*E. bieneusi* MJ13	(1) ITS	MT895460	Red-and-green macaw (*Ara chloropterus*)	
*E. bieneusi* SC02	(3) ITS	MT895459	Great pied hornbill (*Buceros bicomis*)	(2)
			Red-and-green macaw (*Ara chloropterus*)	(1)
*E. bieneusi* SH_ch1	(2) ITS	MT895458*	Cheetah (*Acinonyx jubatus*)	(2)
*E. bieneusi* SH_deer1	(1) ITS	MT895456*	Sika deer (*Cervus Nippon*)	(1)
*E. bieneusi* type IV	(1) ITS	MT895461	Chestnut-fronted macaw (*Ara severa*)	(1)
*E. bieneusi* BEB6-like	(1) ITS	MT895462^a^	Red deer (*Cervus elaphus*)	(1)
*E. bieneusi* MJ17-like	(1) ITS	MT895463^a^	Brown bear (*Ursus arctos pruinosus*)	(1)

*Novel genotypes. ^a^Mixed (indeterminate) genotypes.

**FIGURE 1:**
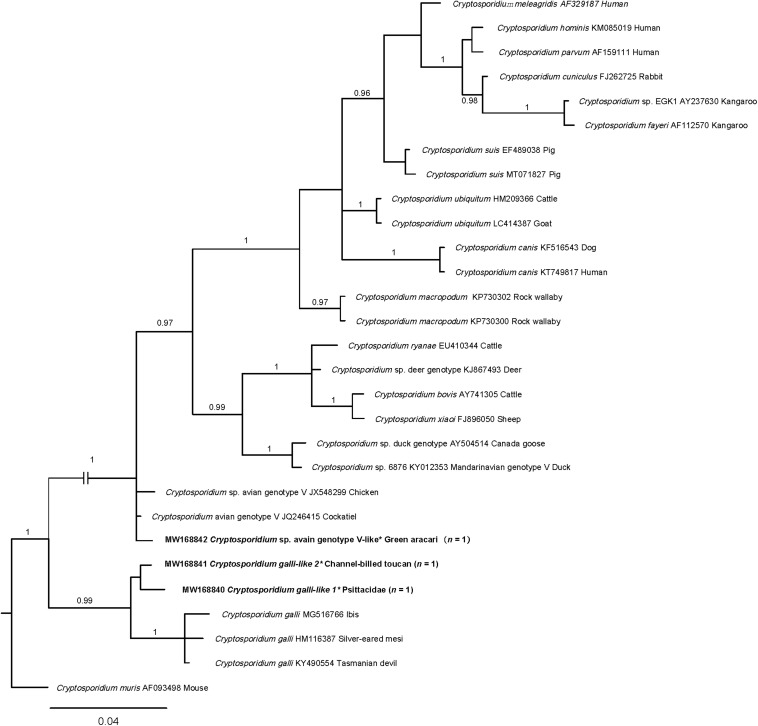
Relationships among *Cryptosporidium* taxa inferred from the phylogenetic analysis of partial small subunit ribosomal rDNA gene (*SSU* rDNA) sequence data by Bayesian inference (BI). Posterior probabilities are indicated at all major nodes. Bold font indicates *Cryptosporidium* species or genotypes characterized from fecal DNA samples in this study. In parentheses are the numbers of samples representing a particular species, genotype, and sequence (GenBank accession numbers indicated). Novel genotypes (^∗^). Scale bar represents the number of substitutions per site. Most clades were strongly supported (*pp* = 0.96–1.00). *pp* < 0.95 was not shown.

The three *SSU* rDNA sequences were aligned with selected representative sequences in particular clades and subjected to the phylogenetic analysis (Figure 1). Genotypes *Cryptosporidium* avian genotype V-like clustered with genotype V with strong statistical support (*pp* = 1). *Cryptosporidium galli*-like 1 and 2 fell in one group and clustered with a clade of *C. galli* (*pp* = 0.99).

### *E. bieneusi* Genotype Characterizations Based on ITS Region

*Enterocytozoon* DNA was specifically detected by nested PCR of ITS in 21 of 182 (11.5%) fecal samples from zoo animals in Zhejiang (3.8%; 2/52) and Shanghai (14.6%; 19/130) (Table 2), including 10 mammal species: Alpaca (*Vicugna pacos*), amur tiger (*Panthera tigris altaica*), brown bear (*Ursus arctos pruinosus*), cheetah (*Acinonyx jubatus*), fallow deer (*Dama dama*), lion (*Panthera leo*), red deer (*Cervus elaphus*), sika deer (*Cervus Nippon*), snub-nosed monkey (*Rhinopithecus* species), tiger (*Panthera tigris tigris*), and three species of birds: Chestnut-fronted macaw (*Ara severa*), great pied hornbill (*Buceros bicomis*), and red-and-green macaw (*Ara chloropterus*) (Table 3).

The 21 ITS amplicons (243 bp) were aligned to reference sequences in the GenBank database, and seven distinct genotypes were identified, including five known (BEB6, D, MJ13, SC02, and type IV) and two novel genotypes (designated SH_ch1 and SH_deer1) (Table 3). Novel genotype SH_ch1 (*n* = 2; from cheetahs) differed by 1 bp (242/243; 99.6%) from the sequence representing genotypes and KIN-1 (GenBank number MT231508). Novel genotype SH_deer1 (*n* = 1; from a sika deer) showed 8-bp (234/242; 99.7%) differences from the sequence with GenBank accession number KF261802. Two ambiguous sequences were derived from two amplicons, each containing multiple genotypes.

**FIGURE 2:**
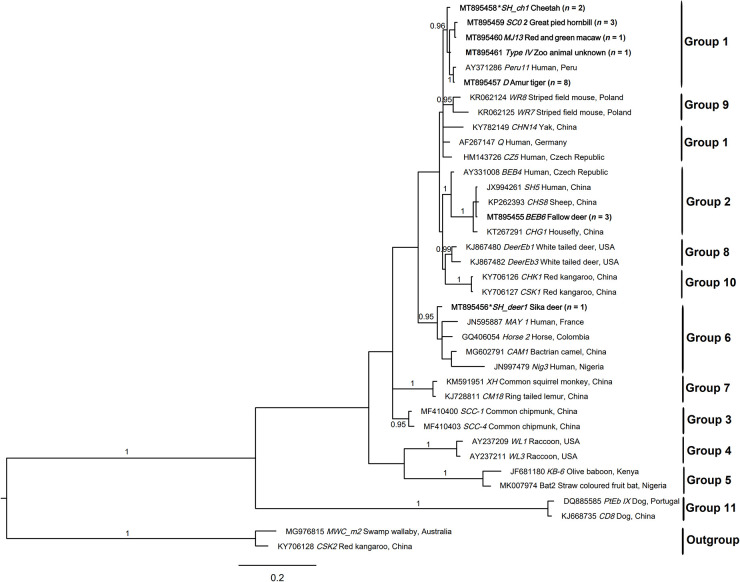
Relationships among the genotypes of *Enterocytozoon bieneusi* recorded in the wildlife in this study inferred from the phylogenetic analysis of sequence data for the internal transcribed spacer (ITS) of nuclear ribosomal DNA by Bayesian inference (BI). Statistically significant posterior probabilities (pps) are indicated on branches. Individual GenBank accession numbers precede genotype designation (in italics) followed by sample and locality descriptions. The *Enterocytozoon bieneusi* genotypes identified and characterized from fecal DNA samples in the present study are indicated in bold type. Clades were assigned group names based on the classification system established by Karim et al. (2015) and Li et al. (2019a). The scale bar represents the number of substitutions per site. The *E. bieneusi* genotypes PtEbIX (DQ885585) and CD8 (KJ668735) from dogs were used as outgroups. All the groups were strongly supported (*pp* = 0.96–1). *pp* < 0.95 were not shown.

The eight ITS sequences representing seven distinct genotypes were aligned with sequences representing 10 groups of *E. bieneusi* and subjected to phylogenetic analysis (Figure 2). Genotypes BEB6, D, MJ13, SC02, SH_ch1, and type IV could be assigned to group 1 (*pp* = 0.96). Novel genotype SH_deer1 clustered with genotypes in group 6 with strong statistical support (*pp* = 0.95).

### *G. duodenalis* Assemblages Identification Based on *tpi* Gene

Sequencing of all *tpi* amplicons identified 48 of 182 (26.4%) individual fecal samples to contain *Giardia* based on direct sequence comparisons, including 8 (15.4%; 8/52) in Zhejiang zoo and 40 (30.8%; 40/130) in Shanghai Wild Animal Park (Table 2). Genetic assemblages A (*n* = 36), C (*n* = 1), and E (*n* = 7) of *G. duodenalis* were characterized, and four amplicons contained mixed indeterminate genotypes. In total, eight distinct sequence types for *tpi* were defined (Table 3), including four representing *Giardia* sub-assemblage A (i.e., one known type from 16 species of wildlife and three novel sequence types from cheetah, fennec fox, lion, and snub-nosed monkey), one novel sequence type from a spotted hyena defined as assemblage C, and two novel distinct sequence types all representing assemblage E from giraffes.

**FIGURE 3:**
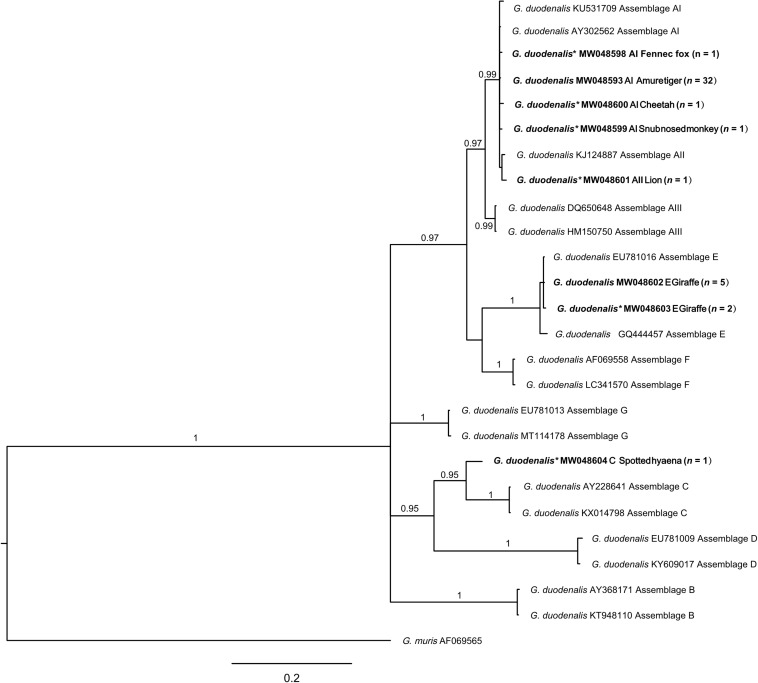
Relationships among *Giardia* taxa inferred from the phylogenetic analysis of partial triose-phosphate isomerase gene (*tpi*) sequence data by Bayesian inference (BI). Posterior probabilities are indicated at all major nodes. Bold font indicates *Giardia* species or genotypes characterized from fecal DNA samples in this study. In parentheses are the numbers of samples representing a particular species, genotype, and sequence (GenBank accession numbers indicated). Novel genotypes (*). Scale bar represents the number of substitutions per site. All groups were strongly supported (*pp* = 0.96–1.00). *pp* < 0.95 were not shown.

The eight distinct *tpi* sequences representing four distinct assemblages or sub-assemblages were aligned with sequences representing *Giardia* assemblages A–G and subjected to the phylogenetic analysis (Figure 3). A novel sequence type (GenBank accession no. MW048604) clustered with assemblage C with strong statistical support (*pp* = 1.00).

## Discussion

The zoonotic enteric pathogens *Cryptosporidium*, *Enterocytozoon*, and *Giardia* have been reported in captive, wild, and zoo animals around the world (Leśniańska et al., 2016; Li N. et al., 2018; Amer et al., 2019). Their ability to spread via contaminated food, water, or direct contact with humans (e.g., zookeeper) poses a risk to public health.

### Cryptosporidium

PCR-based sequencing of all three amplicons from 182 fecal DNA samples (1.6%; 3/182) revealed three operational taxonomic units (OTUs) of *Cryptosporidium* from three birds (channel-billed toucan, green aracari, and an unknown species of Psittacidae). Their *SSU* rDNA sequences were aligned (over a consensus length of 735 positions) with publicly available sequences, representing 14 species and an outgroup *C. muris* (Figure 1). Phylogenetic analyses of *SSU* rDNA data revealed that *Cryptosporidium* avian genotype V-like clustered with the genotypes *C. galli* and *Cryptosporidium* avian genotype V, which are typically found in birds (Xiao et al., 2004), and novel OTUs (genotypes *C. galli*-like 1 and 2 grouped, with strong nodal support (*pp* = 0.99). This analysis clearly showed that *C. galli*-like 1 and 2 represent a new and distinct clade. As the sequence variation (0–1.2%) within novel *C. galli*-like group was substantially less than differences (2.7–3.7%) between *C. galli* group and *C. galli*-like 1 and 2 upon pairwise comparison (Figure 1 and Supplementary Table 2), we propose that the latter two genotypes may represent a novel species of *Cryptosporidium*. However, it should be cautious to draw this conclusion. Definitely, further histological and morphological studies are needed. Sequencing *SSU* rDNA from many more representatives of *Cryptosporidium* to conduct a comprehensive phylogenetic analysis is also required.

### E. bieneusi

*E. bieneusi* was identified in three wildlife fecal DNA samples in Zhejiang zoo (3.8%; 2/52) and 19 in Shanghai Wild Animal Park (14.6%; 19/130), with a total prevalence of 11.5% (21/182). Similarly, Li J. et al. (2015) and Yu et al. (2017) studied the prevalence of *E. bieneusi* in Shanghai wildlife animal park and reported 44.8% (30/67) and 69.1% (38/55), respectively. These cited prevalences are all higher than that in our study; however, they uniquely focused on the populations of non-human primates. By contrast, Li et al. (2016) studied 70 different wildlife species (272 fecal samples) in Chengdu zoo and Bifengxia zoo with prevalences of 10.6% (21/198) and 29.7% (22/74), respectively, both of which are higher than that in Zhejiang zoo, but *E. bieneusi* positivity in Chengdu zoo was lower than that in Shanghai, indicating that *E. bieneusi* might be widespread in Shanghai wild animal park. Internationally, the overall prevalences of *E. bieneusi* in farmed and/or captive wildlife and zoo animals globally ranged from 1.4% in Australia (Zhang et al., 2018c) to 53.3% in China (Yu et al., 2020). The variety of *E. bieneusi* prevalences might be due to host species, health status, and immunity of animals; management; locations; sample size; and environmental factors—season, temperature, sunlight, and humidity.

In total, five known (BEB6, D, MJ13, SC02, and type IV) and two novel genotypes (designated SH_ch1 and SH_deer1) were identified in this study. The predominant genotype here was genotype D (38.1%; 8/21), followed by BEB6 and SC02 (each 14.3%; 3/21), SH_ch1 (9.5%; 2/21), and four other genotypes (each 4.8%; 1/21). Genotype D is frequently identified in humans and nearly 70 species of animals, including birds (Anseriformes, Columbiformes, Falconiformes, Galliformes, Gruiformes, and Passeriformes) and mammals (Artiodactyla, Carnivora, Lagomorpha, Perissodactyla, Primates, and Rodentia) (Zhang, 2019; Zhang et al., 2021), indicating that genotype D has the capability of intra-species transmission. Similarly, genotype BEB6 has also been found in humans and 23 animal species (Zhang, 2019; Zhang et al., 2021), and fallow deer (reported here) is the first record of this genotype. Genotype SC02 was found in human and bear (Wu et al., 2018), giant panda (Li W. et al., 2018), horse (Deng et al., 2016b), Pallas’s squirrel, raccoon (Li et al., 2016), red-bellied tree squirrel (Deng et al., 2016a), rhesus macaque (Zhong et al., 2017), and wild boar (Li et al., 2017); great pied hornbill (*Buceros bicomis*) identified in this study is the first such published record. Similarly, red-and-green macaw (*Ara chloropterus*) is the first host record of genotype MJ13. Predominant genotypes BEB6, D, and SC02 were also found in water samples (Ayed et al., 2012; Li et al., 2012; Huang et al., 2017; Li W. et al., 2018), indicating that they might spread via *E. bieneusi* spores–contaminated water.

Phylogenetic analyses revealed that novel genotype SH_deer1 clustered with genotypes CAM1 (camel), horse 2 (horse), MAY 1 (human), and Nig3 (human), falling into group 6. Previously, genotypes in this group were predominantly found in animals. Thus, group 6 was typically considered as the host-adapted group. However, with more genotypes from this group identified in humans (Akinbo et al., 2012; Qi et al., 2018), demonstrating that group 6 revealed zoonotic potential. Additionally, we have also created a phylogeny using all nearly 600 unique genotypes from all published studies employing complete *ITS* sequences, with the aim of assessing the relationships of the genotypes and the validity of groups (Zhang et al., 2021), proving the zoonotic potential of group 6. The overall results indicate that wildlife carrying zoonotic genotypes have the capacity to transmit from them to humans.

### G. duodenalis

In the present study, 48 wildlife tested positive for *G. duodenalis* with a total prevalence of 26.37% (48/182), which was higher than that of *Cryptosporidium* (1.6%; 3/182) and *E. bieneusi* (11.5%; 21/182), indicating that *G. duodenalis* is more widely spread than the other two microbes. The prevalences of *G. duodenalis* in Shanghai Wild Animal Park and in Zhejiang were 30.8% (40/130) and 15.4% (8/52), respectively, both of which were higher than that in a number of studies of *G. duodenalis* globally (Matsubayashi et al., 2005; Lallo et al., 2009; Beck et al., 2011b; Majewska et al., 2012; Oates et al., 2012; Aghazadeh et al., 2015; Reboredo-Fernández et al., 2015; Adriana et al., 2016; Mynarova et al., 2016; Mateo et al., 2017; Helmy et al., 2018). Additionally, the prevalence of *G. duodenalis* in wild animals worldwide ranged from 1.1% in zoo in Japan (Matsubayashi et al., 2005) to 29.0% in Zagreb zoo in Croatia (Beck et al., 2011a); 30.8% (40/130) here in wildlife in Shanghai is the highest prevalence around the world. The overall results indicate relatively high *G. duodenalis* infections in zoo animals in this study. However, it cannot be entirely excluded that *G. duodenalis* cysts might only pass through the gastrointestinal tract (pseudoparasitism), as identification of *G. duodenalis* DNA from fecal samples is not a direct evidence of infection.

In total, three assemblages A, C, and E of *G. duodenalis* were characterized. Zoonotic assemblage A is predominant (75%; 36/48) in this study, followed by genotype E (14.58%; 7/48) and C (2.08%; 1/48). Genotype A has been reported in humans and a large number of animal species with the capacity of cross-species transmission (Ryan and Zahedi, 2019). In this study, assemblage E was mostly identified in giraffe, except for one positivity in kangaroo. This is the first time that kangaroo was recorded in the *G. duodenalis* assemblage E. This assemblage has been mainly reported in hoofed animals, but it was also detected in human specimens in Brazil (Fantinatti et al., 2016), Egypt (Foronda et al., 2008), and Australia (Zahedi et al., 2017), posing less risk to public health. Phylogenetically, the novel *tpi* sequence found in spotted hyena (*Crocuta crocuta*) clustered with assemblage C (Figure 3), which has been frequently reported in canids and occasionally reported in humans (Hopkins et al., 1997; Monis et al., 1998). The overall results indicate that zoo animals can harbor zoonotic *G. duodenalis* and potentially act as a host reservoir for human infections of giardiasis.

## Conclusion

Exploring the genetic composition of *Cryptosporidium* species *E. bieneusi* and *G. duodenalis* populations in animals and humans is important for understanding transmission patterns of enteric disease and for its prevention and control. By conducting the present molecular-phylogenetic investigation of three pathogens target sequences derived from fecal samples (*n* = 182) from zoo animals in China, we found (phylogenetically) a novel species of *Cryptosporidium*. We also identified genotypes or assemblages (*E. bieneusi*: BEB6, D, MJ13, SC02, SH_ch1, SH_deer1, and type IV; *G. duodenalis*: A, C, and E), all of which have zoonotic potential. The overall results indicate that wildlife carrying zoonotic *E. bieneusi* and *G. duodenalis* can potentially transmit the pathogens to humans, thus posting a public health risk.

## Data Availability Statement

The datasets generated for this study can be found in GenBank under the accession numbers, *Cryptosporidium*: MW168840-MW168842; *E. bieneusi*: MT895455-MT895461 *and G. duodenalis*: MW048593-MW048601.

## Ethics Statement

Sample collections were carried out by colleges from the Shanghai Wild Animal Park and Zhejiang Zoo. Animals were handled in accordance with the Animal Ethics Procedures and Guidelines of the People’s Republic of China.

## Author Contributions

YZ, RM, LY, ZC, YF, XC, YH, and HG: sample collection. YZ and LY: designed the study and performed the experiments. YZ: analysis and interpretation and wrote the manuscript. ZC: review the draft and supervision of project. All authors read and approved the final version of the manuscript.

## Conflict of Interest

The authors declare that the research was conducted in the absence of any commercial or financial relationships that could be construed as a potential conflict of interest.

## Publisher’s Note

All claims expressed in this article are solely those of the authors and do not necessarily represent those of their affiliated organizations, or those of the publisher, the editors and the reviewers. Any product that may be evaluated in this article, or claim that may be made by its manufacturer, is not guaranteed or endorsed by the publisher.

## Supplementary Material

Supplementary Table 1 The information regarding fecal samples (sample codes are given) collected from various species of wildlife (host and Latin name are given) located in Zhejiang and Shanghai from May 2018 to August 2020.

Supplementary Table 2 Pairwise comparison of sequence differences in the small subunit of nuclear ribosomal RNA gene (*SSU* rDNA; 735 bp) among *Cryptosporidium* species from samples tested in the present study (cf. Figure 1).
