# Corrigendum: Wildlife is a potential source of human infections of *Enterocytozoon bieneusi* and *Giardia duodenalis* in southeastern China

**DOI:** 10.3389/fmicb.2022.1034984

**Published:** 2022-09-22

**Authors:** Yan Zhang, Rongsheng Mi, Lijuan Yang, Haiyan Gong, Chunzhong Xu, Yongqi Feng, Xinsheng Chen, Yan Huang, Xiangan Han, Zhaoguo Chen

**Affiliations:** ^1^Key Laboratory of Animal Parasitology of Ministry of Agriculture, Laboratory of Quality and Safety Risk Assessment for Animal Products on Biohazards (Shanghai) of Ministry of Agriculture, Shanghai Veterinary Research Institute, Chinese Academy of Agricultural Sciences, Shanghai, China; ^2^Shanghai Wild Animal Park, Shanghai, China

**Keywords:** *Cryptosporidium*, *Enterocytozoon bieneusi*, *Giardia duodenalis*, genotypes, wildlife, prevalence, zoonotic potential

In the published article, there was an error in Figure 2 as published. The legend of Figure 2 is correct, but Figure 2 is exactly the same as Figure 3, which is wrong. The corrected Figure 2 and its caption appear below.

**Figure 2:**
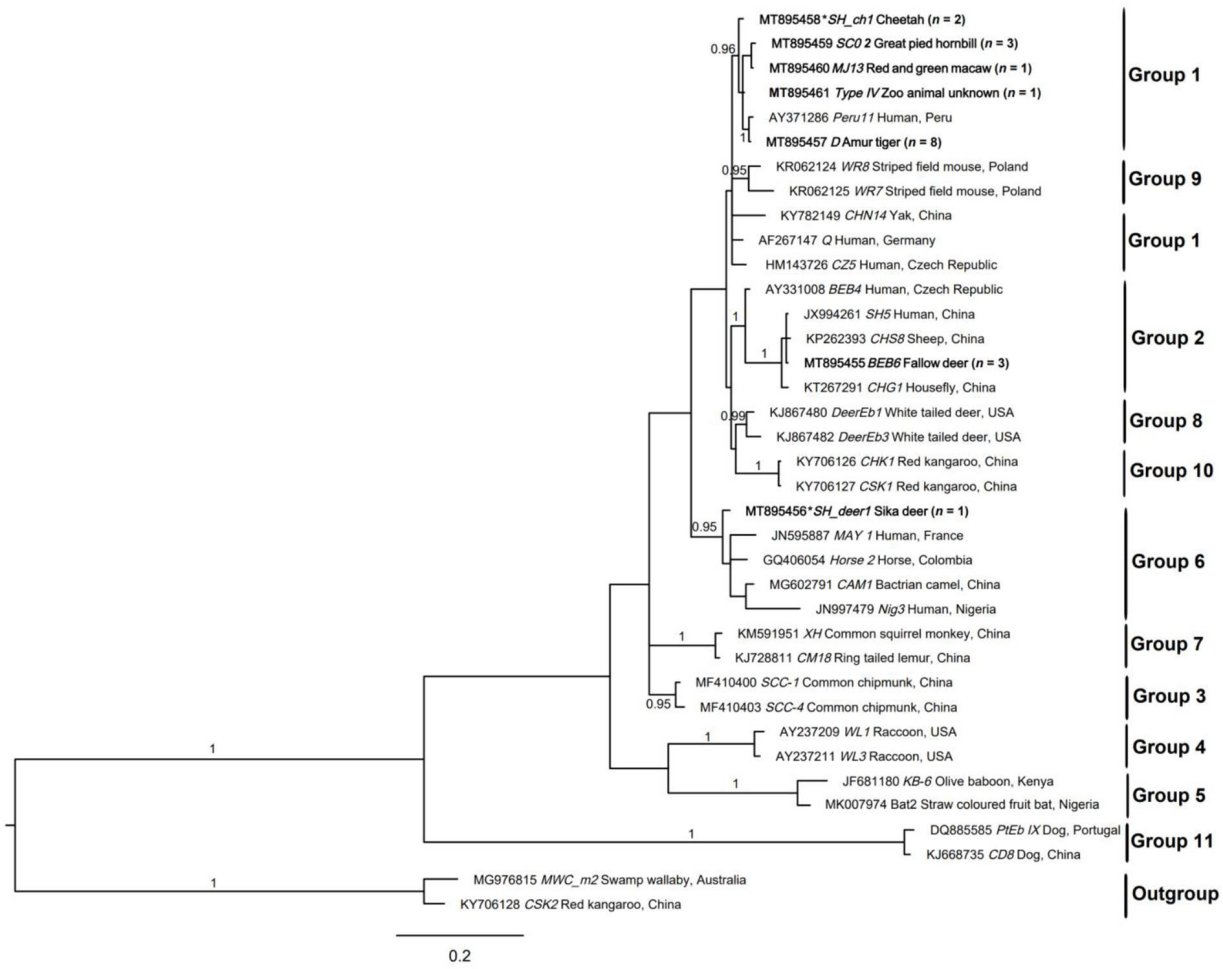
Relationships among the genotypes of *Enterocytozoon bieneusi* recorded in the wildlife in this study inferred from the phylogenetic analysis of sequence data for the internal transcribed spacer (ITS) of nuclear ribosomal DNA by Bayesian inference (BI). Statistically significant posterior probabilities (pps) are indicated on branches. Individual GenBank accession numbers precede genotype designation (in italics) followed by sample and locality descriptions. The *Enterocytozoon bieneusi* genotypes identified and characterized from fecal DNA samples in the present study are indicated in bold type. Clades were assigned group names based on the classification system established by Karim et al. (2015) and Li et al. (2019a). The scale bar represents the number of substitutions per site. The *E. bieneusi* genotypes PtEbIX (DQ885585) and CD8 (KJ668735) from dogs were used as outgroups. All the groups were strongly supported (*pp* = 0.96–1). *pp* < 0.95 were not shown.

The authors apologize for this error and state that this does not change the scientific conclusions of the article in any way. The original article has been updated.

## Publisher's note

All claims expressed in this article are solely those of the authors and do not necessarily represent those of their affiliated organizations, or those of the publisher, the editors and the reviewers. Any product that may be evaluated in this article, or claim that may be made by its manufacturer, is not guaranteed or endorsed by the publisher.
