# Supplementary material for: Wildlife Is a Potential Source of Human Infections of Enterocytozoon bieneusi and Giardia duodenalis in Southeastern China
Source: Front Microbiol. 2021 Aug 10;12:692837. doi: 10.3389/fmicb.2021.692837 (PMC8383182; doi:10.3389/fmicb.2021.692837)
Supplement: Supplementary Table 1 — The information regarding fecal samples (sample codes are given) collected from various species of wildlife (host and Latin name are given) located in Zhejiang and Shanghai from May 2018 to August 2020. [file Table_1.DOCX]

**Table S1.** The information regarding faecal samples (sample codes are given) collected from various species of wildlife (host and Latin name are given) located in Zhejiang and Shanghai from May 2018 to August 2020

| **Sample code** | **Host** | **Latin name** | **Year** | **Season** | **Loca-tion** |
| --- | --- | --- | --- | --- | --- |
| YZZ001 | Fallow deer | *Dama dama* | 2020 | Winter | SH |
| YZZ002 | Fallow deer | *Dama dama* | 2020 | Winter | SH |
| YZZ003* | Fallow deer | *Dama dama* | 2020 | Winter | SH |
| YZZ004 | Yak | *Bos mutus* | 2020 | Winter | SH |
| YZZ005 | Yak | *Bos mutus* | 2020 | Winter | SH |
| YZZ006 | Yak | *Bos mutus* | 2020 | Winter | SH |
| YZZ007 | Lama | *Lama glama* | 2020 | Winter | SH |
| YZZ008 | Lama | *Lama glama* | 2020 | Winter | SH |
| YZZ009 | Lama | *Lama glama* | 2020 | Winter | SH |
| YZZ010 | Waterbuffalo | *Bubalus arnee* | 2020 | Winter | SH |
| YZZ011 | Waterbuffalo | *Bubalus arnee* | 2020 | Winter | SH |
| YZZ012 | Waterbuffalo | *Bubalus arnee* | 2020 | Winter | SH |
| YZZ013* | Red deer | *Cervus elaphus* | 2020 | Winter | SH |
| YZZ014* | Red deer | *Cervus elaphus* | 2020 | Winter | SH |
| YZZ015 | Red deer | *Cervus elaphus* | 2020 | Winter | SH |
| YZZ016 | Pony | *Equus ferus caballus* | 2020 | Winter | SH |
| YZZ017 | Pony | *Equus ferus caballus* | 2020 | Winter | SH |
| YZZ018 | Pony | *Equus ferus caballus* | 2020 | Winter | SH |
| YZZ019 | Greater one-horned rhinoceros | *Rhinoceros unicornis* | 2020 | Winter | SH |
| YZZ020 | Greater one-horned rhinoceros | *Rhinoceros unicornis* | 2020 | Winter | SH |
| YZZ021 | Greater one-horned rhinoceros | *Rhinoceros unicornis* | 2020 | Winter | SH |
| YZZ022 | Central American tapir | *Tapirus bairdii* | 2020 | Winter | SH |
| YZZ023 | Central American tapir | *Tapirus bairdii* | 2020 | Winter | SH |
| YZZ024 | Central American tapir | *Tapirus bairdii* | 2020 | Winter | SH |
| YZZ025* | Giraffe | *Giraffa camelopardalis* | 2020 | Winter | SH |
| YZZ026* | Giraffe | *Giraffa camelopardalis* | 2020 | Winter | SH |
| YZZ027* | Giraffe | *Giraffa camelopardalis* | 2020 | Winter | SH |
| YZZ028* | Hippopotamus | *Hippopotamus amphibius* | 2020 | Winter | SH |
| YZZ029 | Hippopotamus | *Hippopotamus amphibius* | 2020 | Winter | SH |
| YZZ030 | Hippopotamus | *Hippopotamus amphibius* | 2020 | Winter | SH |
| YZZ031 | Golden takin | *Budorcas taxicolor bedfordi* | 2020 | Winter | SH |
| YZZ032 | Golden takin | *Budorcas taxicolor bedfordi* | 2020 | Winter | SH |
| YZZ033* | Golden takin | *Budorcas taxicolor bedfordi* | 2020 | Winter | SH |
| YZZ034 | Giant Eland | *Tragelaphus derbianus* | 2020 | Winter | SH |
| YZZ035* | Giant Eland | *Tragelaphus derbianus* | 2020 | Winter | SH |
| YZZ036 | Giant Eland | *Tragelaphus derbianus* | 2020 | Winter | SH |
| YZZ037 | Burchell's zebra | *Equus burchellii* | 2020 | Winter | SH |
| YZZ038 | Burchell's zebra | *Equus burchellii* | 2020 | Winter | SH |
| YZZ039 | Burchell's zebra | *Equus burchellii* | 2020 | Winter | SH |
| YZZ040* | Alpaca | *Vicugna pacos* | 2020 | Winter | SH |
| YZZ041 | Alpaca | *Vicugna pacos* | 2020 | Winter | SH |
| YZZ042* | Alpaca | *Vicugna pacos* | 2020 | Winter | SH |
| YZZ043* | Sika deer | *Cervus nippon* | 2020 | Winter | SH |
| YZZ044* | Sika deer | *Cervus nippon* | 2020 | Winter | SH |
| YZZ045 | Sika deer | *Cervus nippon* | 2020 | Winter | SH |
| YZZ046 | Ostrich | *Struthio camelus* | 2020 | Winter | SH |
| YZZ047* | Ostrich | *Struthio camelus* | 2020 | Winter | SH |
| YZZ048* | Ostrich | *Struthio camelus* | 2020 | Winter | SH |
| YZZ049 | Oriental pied hornbill | *Anthracoceros albirostris* | 2020 | Winter | SH |
| YZZ050 | Oriental pied hornbill | *Anthracoceros albirostris* | 2020 | Winter | SH |
| YZZ051 | Oriental pied hornbill | *Anthracoceros albirostris* | 2020 | Winter | SH |
| YZZ052 | Rheas | *Rhea americana* | 2020 | Winter | SH |
| YZZ053 | Rheas | *Rhea americana* | 2020 | Winter | SH |
| YZZ054 | Rheas | *Rhea americana* | 2020 | Winter | SH |
| YZZ055 | Black-necked Crane | *Grus nigricollis* | 2020 | Winter | SH |
| YZZ056* | Black-necked Crane | *Grus nigricollis* | 2020 | Winter | SH |
| YZZ057* | Black-necked Crane | *Grus nigricollis* | 2020 | Winter | SH |
| YZZ058* | Fennec fox | *Vulpes zerda* | 2020 | Winter | SH |
| YZZ059* | Fennec fox | *Vulpes zerda* | 2020 | Winter | SH |
| YZZ060 | Fennec fox | *Vulpes zerda* | 2020 | Winter | SH |
| YZZ061* | Lion | *Panthera leo* | 2020 | Winter | SH |
| YZZ062* | Lion | *Panthera leo* | 2020 | Winter | SH |
| YZZ063* | Lion | *Panthera leo* | 2020 | Winter | SH |
| YZZ064* | Amur tiger | *Panthera tigris altaica* | 2020 | Winter | SH |
| YZZ065* | Amur tiger | *Panthera tigris altaica* | 2020 | Winter | SH |
| YZZ066* | Amur tiger | *Panthera tigris altaica* | 2020 | Winter | SH |
| YZZ067* | Cheetahs | *Acinonyx jubatus* | 2020 | Winter | SH |
| YZZ068* | Cheetah | *Acinonyx jubatus* | 2020 | Winter | SH |
| YZZ069* | Cheetah | *Acinonyx jubatus* | 2020 | Winter | SH |
| YZZ070 | Tiger | *Panthera tigris tigris* | 2020 | Winter | SH |
| YZZ071* | Tiger | *Panthera tigris tigris* | 2020 | Winter | SH |
| YZZ072* | Tiger | *Panthera tigris tigris* | 2020 | Winter | SH |
| YZZ073 | Asian elephant | *Elephas maximus Linnaeus* | 2020 | Winter | SH |
| YZZ074 | Asian elephant | *Elephas maximus Linnaeus* | 2020 | Winter | SH |
| YZZ075 | Asian elephant | *Elephas maximus Linnaeus* | 2020 | Winter | SH |
| YZZ076 | Peafowl | *Pavo cristatus* | 2020 | Winter | SH |
| YZZ077* | Peafowl | *Pavo cristatus* | 2020 | Winter | SH |
| YZZ078* | Peafowl | *Pavo cristatus* | 2020 | Winter | SH |
| YZZ079 | Sun Conure | *Aratinga solstitialis* | 2020 | Winter | SH |
| YZZ080* | Sun Conure | *Aratinga solstitialis* | 2020 | Winter | SH |
| YZZ081 | Sun Conure | *Aratinga solstitialis* | 2020 | Winter | SH |
| YZZ082 | Crested Mynas | *Acridotheres cristatellus* | 2020 | Winter | SH |
| YZZ083 | Crested Mynas | *Acridotheres cristatellus* | 2020 | Winter | SH |
| YZZ084 | Crested Mynas | *Acridotheres cristatellus* | 2020 | Winter | SH |
| YZZ085 | Malabar pied hornbill | *Anthracoceros coronatus* | 2020 | Winter | SH |
| YZZ086* | Malabar pied hornbill | *Anthracoceros coronatus* | 2020 | Winter | SH |
| YZZ087 | Malabar pied hornbill | *Anthracoceros coronatus* | 2020 | Winter | SH |
| YZZ088 | Scarlet macaw | *Ara macao* | 2020 | Winter | SH |
| YZZ089 | Scarlet macaw | *Ara macao* | 2020 | Winter | SH |
| YZZ090* | Scarlet macaw | *Ara macao* | 2020 | Winter | SH |
| YZZ091* | Blue-headed macaw | *Propyrrhura couloni* | 2020 | Winter | SH |
| YZZ092* | Blue-headed macaw | *Propyrrhura couloni* | 2020 | Winter | SH |
| YZZ093* | Blue-headed macaw | *Propyrrhura couloni* | 2020 | Winter | SH |
| YZZ094 | Blue-and-yellow macaw | *Ara ararauna* | 2020 | Winter | SH |
| YZZ095 | Blue-and-yellow macaw | *Ara ararauna* | 2020 | Winter | SH |
| YZZ096 | Blue-and-yellow macaw | *Ara ararauna* | 2020 | Winter | SH |
| YZZ097 | Black bear | *Ursus americanus luteolus* | 2020 | Winter | SH |
| YZZ098 | Black bear | *Ursus americanus luteolus* | 2020 | Winter | SH |
| YZZ099 | Black bear | *Ursus americanus luteolus* | 2020 | Winter | SH |
| YZZ100 | Brown bear | *Ursus arctos pruinosus* | 2020 | Winter | SH |
| YZZ101 | Brown bear | *Ursus arctos pruinosus* | 2020 | Winter | SH |
| YZZ102 | Brown bear | *Ursus arctos pruinosus* | 2020 | Winter | SH |
| YZZ103* | Great pied hornbill | *Buceros bicomis* | 2020 | Winter | SH |
| YZZ104* | Great pied hornbill | *Buceros bicomis* | 2020 | Winter | SH |
| YZZ105* | Great pied hornbill | *Buceros bicomis* | 2020 | Winter | SH |
| YZZ106* | Red-and-green macaw | *Ara chloropterus* | 2020 | Winter | SH |
| YZZ107* | Red-and-green macaw | *Ara chloropterus* | 2020 | Winter | SH |
| YZZ108 | Red-bellied macaw | Orthopsittaca manilata | 2018 | Spring | ZJ |
| YZZ109 | Red-shouldered macaw | Diopsittaca nobilis | 2018 | Spring | ZJ |
| YZZ110* | Psittacidae | NA | 2018 | Spring | ZJ |
| YZZ111* | Channel-billed toucan | *Ramphastos vitellinus* | 2018 | Spring | ZJ |
| YZZ112* | Green aracari | *Pteroglossus viridis* | 2018 | Spring | ZJ |
| YZZ113 | Black-necked aracari | *Pteroglossus aracari* | 2018 | Spring | ZJ |
| YZZ114 | Guianan toucanet | *Selenidera culik* | 2018 | Spring | ZJ |
| YZZ115 | Black-headed caique | *Pionites melanocephalus* | 2018 | Spring | ZJ |
| YZZ116* | Chestnut-fronted macaw | *Ara severa* | 2018 | Spring | ZJ |
| YZZ117 | Spotted towhee | *Pipilo maculatus* | 2018 | Spring | ZJ |
| YZZ118 | Red-fan Parrot | *Deroptyus accipitrinus* | 2018 | Spring | ZJ |
| YZZ119 | Serval | *Leptailurus serval* | 2018 | Spring | ZJ |
| YZZ120 | Lion | *Panthera leo* | 2018 | Spring | ZJ |
| YZZ121 | Lion | *Panthera leo* | 2018 | Spring | ZJ |
| YZZ122 | Lion | *Panthera leo* | 2018 | Spring | ZJ |
| YZZ123 | Lion | *Panthera leo* | 2018 | Spring | ZJ |
| YZZ124 | Lion | *Panthera leo* | 2018 | Spring | ZJ |
| YZZ125 | Puma | *Puma concolor* | 2018 | Spring | ZJ |
| YZZ126 | Puma | *Puma concolor* | 2018 | Spring | ZJ |
| YZZ127 | Giraffe | *Giraffa camelopardalis* | 2018 | Spring | ZJ |
| YZZ128* | Giraffe | *Giraffa camelopardalis* | 2018 | Spring | ZJ |
| YZZ129 | Giraffe | *Giraffa camelopardalis* | 2018 | Spring | ZJ |
| YZZ130* | Giraffe | *Giraffa camelopardalis* | 2018 | Spring | ZJ |
| YZZ131* | Giraffe | *Giraffa camelopardalis* | 2018 | Spring | ZJ |
| YZZ132 | Giraffe | *Giraffa camelopardalis* | 2018 | Spring | ZJ |
| YZZ133* | Giraffe | *Giraffa camelopardalis* | 2018 | Spring | ZJ |
| YZZ134* | Giraffe | *Giraffa camelopardalis* | 2018 | Spring | ZJ |
| YZZ135* | Giraffe | *Giraffa camelopardalis* | 2018 | Spring | ZJ |
| YZZ136 | Giraffe | *Giraffa camelopardalis* | 2018 | Spring | ZJ |
| YZZ137 | Giraffe | *Giraffa camelopardalis* | 2018 | Spring | ZJ |
| YZZ138 | Giraffe | *Giraffa camelopardalis* | 2018 | Spring | ZJ |
| YZZ139 | Giraffe | *Giraffa camelopardalis* | 2018 | Spring | ZJ |
| YZZ140 | Giraffe | *Giraffa camelopardalis* | 2018 | Spring | ZJ |
| YZZ141 | Giraffe | *Giraffa camelopardalis* | 2018 | Spring | ZJ |
| YZZ142 | Giraffe | *Giraffa camelopardalis* | 2018 | Spring | ZJ |
| YZZ143 | Giraffe | *Giraffa camelopardalis* | 2018 | Spring | ZJ |
| YZZ144 | Giraffe | *Giraffa camelopardalis* | 2018 | Spring | ZJ |
| YZZ145* | Kangaroo | *Macropus* spp. | 2018 | Spring | ZJ |
| YZZ146 | Kangaroo | *Macropus* spp. | 2018 | Spring | ZJ |
| YZZ147 | Kangaroo | *Macropus* spp. | 2018 | Spring | ZJ |
| YZZ148 | Kangaroo | *Macropus* spp. | 2018 | Spring | ZJ |
| YZZ149 | Kangaroo | *Macropus* spp. | 2018 | Spring | ZJ |
| YZZ150 | Kangaroo | *Macropus* spp. | 2018 | Spring | ZJ |
| YZZ151 | Kangaroo | *Macropus* spp. | 2018 | Spring | ZJ |
| YZZ152 | Kangaroo | *Macropus* spp. | 2018 | Spring | ZJ |
| YZZ153 | Burchell's zebra | *Equus burchellii* | 2018 | Spring | ZJ |
| YZZ154* | Spotted hyaena | *Crocuta crocuta* | 2018 | Spring | ZJ |
| YZZ155 | Camel | *Camelus* spp. | 2018 | Spring | ZJ |
| YZZ156 | Peafowl | *Pavo cristatus* | 2018 | Spring | ZJ |
| YZZ157* | Brown bear | *Ursus arctos pruinosus* | 2018 | Spring | ZJ |
| YZZ158 | Sika deer | *Cervus nippon* | 2018 | Spring | ZJ |
| YZZ159 | Ostrich | *Struthio camelus* | 2018 | Spring | ZJ |
| YZZ160 | Hylobatidae | NA | 2020 | Summer | SH |
| YZZ161 | Hylobatidae | NA | 2020 | Summer | SH |
| YZZ162* | Snub-nosed monkey | *Rhinopithecus* *roxellana* | 2020 | Summer | SH |
| YZZ163 | Snub-nosed monkey | *Rhinopithecus* *roxellana* | 2020 | Summer | SH |
| YZZ164* | Snub-nosed monkey | *Rhinopithecus roxellana* | 2020 | Summer | SH |
| YZZ165 | Snub-nosed monkey | *Rhinopithecus* *roxellana* | 2020 | Summer | SH |
| YZZ166 | Snub-nosed monkey | *Rhinopithecus* *roxellana* | 2020 | Summer | SH |
| YZZ167* | Snub-nosed monkey | *Rhinopithecus roxellana* | 2020 | Summer | SH |
| YZZ168 | Snub-nosed monkey | *Rhinopithecus* *roxellana* | 2020 | Summer | SH |
| YZZ169* | Snub-nosed monkey | *Rhinopithecus* *roxellana* | 2020 | Summer | SH |
| YZZ170 | Snub-nosed monkey | *Rhinopithecus* *roxellana* | 2020 | Summer | SH |
| YZZ171 | Snub-nosed monkey | *Rhinopithecus roxellana* | 2020 | Summer | SH |
| YZZ172 | Snub-nosed monkey | *Rhinopithecus* *roxellana* | 2020 | Summer | SH |
| YZZ173* | Snub-nosed monkey | *Rhinopithecus* *roxellana* | 2020 | Summer | SH |
| YZZ174 | Snub-nosed monkey | *Rhinopithecus roxellana* | 2020 | Summer | SH |
| YZZ175 | Snub-nosed monkey | *Rhinopithecus* *roxellana* | 2020 | Summer | SH |
| YZZ176* | Snub-nosed monkey | *Rhinopithecus* *roxellana* | 2020 | Summer | SH |
| YZZ177 | Snub-nosed monkey | *Rhinopithecus roxellana* | 2020 | Summer | SH |
| YZZ178* | Snub-nosed monkey | *Rhinopithecus* *roxellana* | 2020 | Summer | SH |
| YZZ179 | Snub-nosed monkey | *Rhinopithecus* *roxellana* | 2020 | Summer | SH |
| YZZ180 | Snub-nosed monkey | *Rhinopithecus roxellana* | 2020 | Summer | SH |
| YZZ181 | Snub-nosed monkey | *Rhinopithecus* *roxellana* | 2020 | Summer | SH |
| YZZ182* | Snub-nosed monkey | *Rhinopithecus* *roxellana* | 2020 | Summer | SH |

SH = Shanghai. ZJ = Zhejiang. * = test-positivity.
